# The Top-Cited Articles With a Focus on Barrett's Esophagus: A Bibliometric Analysis

**DOI:** 10.3389/fsurg.2022.743274

**Published:** 2022-02-17

**Authors:** Peiling Gan, Wentao Fan, Hailong Zhang, Chunyu Zhong, Huifang Xia, Muhan Lü, Xian Zhou, Xiaowei Tang

**Affiliations:** ^1^Department of Gastroenterology, Affiliated Hospital of Southwest Medical University, Luzhou, China; ^2^Digestive Endoscopy Department and General Surgery Department, The First Affiliated Hospital With Nanjing Medical University and Jiangsu Province Hospital, Nanjing, China

**Keywords:** top-cited, Barrett's esophagus, bibliometric analysis, visualization, premalignant

## Abstract

**Background:**

Because the number of published literatures with a focus on Barrett's esophagus (BE) that researchers must be familiar with has quickly increased in recent years, the significance of selective searching and summarization of bibliometrics is also increasing. It is, thus, very important to find a method that can quickly and effectively search the most influential medical science articles. Therefore, the objective of this study was to use bibliometric analysis to assess and characterize the most influential articles involving BE research.

**Methods:**

Publications on BE research were retrieved from the Web of Science Core Collection using the term “Barrett's esophagus.” Microsoft Excel 2016 and VOSviewer were used to further analyzed each article's citation number, title, journal, country, organization, category, and authorship.

**Results:**

On 14 June 2020, 5,389 records of BE research published until 2020 were retrieved. The citation number of the top 100 most-cited articles ranged from 208 to 824. *Gastroenterology* published 29 articles, which accounted for the largest number of top 100 articles (29%); however, among the top 500 most-cited articles, the *American Journal of Gastroenterology* published the largest number. Of the top-cited articles, the USA was by far the leading country in BE research and contributed most of the articles (*n* = 72). Among the academic institutions that produced the top 100 most-cited articles, the University of Washington (*n* = 12) was dominant. Sharma Prateek (*n* = 6) authored the largest number of most-cited articles. The USA contributed the most articles per year, and the time trend of the number of top 500 articles increased by 38-fold between 1987 and 2000. “Adenocarcinoma,” “high-grade dysplasia,” “cancer,” “diagnosis,” and “dysplasia” were the most influential keywords.

**Conclusions:**

This study not only presents a historical perspective but also facilitated the recognition of the significant advances in this area by researchers. Furthermore, the current study serves as a guide in decision clinical practice decision-making and provides a valuable reference for further research.

## Introduction

Finding a method that can quickly and effectively search the most influential studies is a common problem. Indeed, no such method has been established. The quantity of literature that researchers must be familiar with to acquire advanced information is rapidly increasing. Besides, the flow of medicine varies from minute-to-minute, medical knowledge is changing, and being redefined continuously, which makes it more difficult to access information that is needed. In response to these difficulties, the consequence of a search method and bibliometric summary recognized by researchers is expanding. As the widespread availability of access to the Internet increases, bibliometric analysis has been implemented as a method that reflects the value of an article.

BE, a metaplastic change involving the lining of the distal esophageal epithelium, is a premalignant condition for esophageal adenocarcinoma, the morbidity of which is on the rise among western countries. The early use of endoscopic therapy is significant for the identification and treatment of BE (1–5). Although endoscopic recognition of dysplasia in BE is difficult, experience in recognition of early neoplastic lesions increases the detection of early neoplastic lesions. In addition, dysplasia, which is a premalignant condition (also referred to as intraepithelial neoplasia), is an important marker for more severe pathologic changes (6). Because of the poor prognosis, BE has attracted the attention of researchers and clinicians worldwide. Many highly cited articles have been analyzed BE in many ways, such as diagnosis, classification, treatment, and prognosis. Bibliometric analysis has been widely used in other fields, such as rheumatology (7), gastric diseases (8), hand and wrist surgery (9), and nuclear medicine (10), to explore developmental trends in various fields through quantitative analysis in the scientific literature. This method facilitates researchers to understand the scope of research topics and predict future directions. Thus, we conducted an analytic study of published articles on BE research using bibliometric data, aimed to describe the scientific outputs of BE research to determine research trends and hotspots, guide researchers' future work.

## Methods

### Search Strategies

We performed a bibliometric analysis on BE. In this retrospective study, we selected articles that were indexed in the representative Web of Science Core Collection (WoSCC) database as the data source. We carried out a study of the top 100 cited articles and top 500 cited articles using the term “Barrett's esophagus”. Only original articles and reviews were included in top 100. The search strategy used in the database was designed on 14 June 2020.

### Inclusion and Exclusion Criteria

Publications indexed in the WoSCC were included in this analysis. Then, we excluded the irrelevant publications from all of the top 100 most frequently cited articles by reviewing the titles and abstracts to identify the relevance to BE. If necessary, we read the full text to determine its relevance and whether the article should be excluded. There were no language, publication year, or web of science category restrictions.

### Statistical Analysis

Microsoft Excel 2016, VOSviewer 1.6.11 (11), and bibliometrics online analysis platform (12) were used to comb the related information and further analyze the retrieved data. We carried out an analysis of the following characteristics: (a) the number of citations; (b) title; (c) journal name with the Eigenfactor^TM^ score (EF); (d) year of publication; (e) authorship; (f) type of research; and (g) article type.

The consequence of a journal is characterized by EF, which is an alternative metric for indicating the authority and influence of journals (13). The EF of a journal was calculated to provide a general influence in the scientific community. Unlike the 5-year journal Impact Factor, the EF excludes self-citation and assigns weight to each earned citation. This score is scaled, thus the scores of all journals in JCR sum to 100. For instance, if a journal has an EF of 1.0, this journal has 1% of the total influence of all publications (14).

All publications were retrieved and ranked in descending order based on the number of citations. All of the initial outcomes from the studies, which are the narrative synthesis of data, were extracted from WoSCC using a pre-designed Microsoft Excel template. VOSviewer is a professional software used for assisting trends in scientific research by analyzing and visualizing a bibliometric network. Network analysis was carried out to identify the most influential keywords, authors, and countries among the top 100 most frequently cited articles using VOSviewer version 1.6.11.

## Results

### WoSCC Bibliometrics

Five thousand three hundred and eighty nine records of BE research published until 2020 were retrieved. Table 1 lists the top 10 cited articles from the WoSCC bibliometrics on BE research, including detailed information in descending order based on the number of citations. The supporting information of the top 100 most cited articles is listed in Supplementary Table S1 in descending order based on the number of citations. The number of citations in the top 100 most frequently cited WoSCC articles on BE research ranged from 208 to 824. The publication year of the top100 articles ranged from 1987 to 2016, and the articles were published in 27 different journals. Among these journals, *Gastroenterology* published 27 articles, which accounted for the largest number of the top 100 articles (27%), with the fourth EF in 2019 (0.132) among the top 10 journals. Followed by the *American Journal of Gastroenterology* (17%), *Gastrointestinal Endoscopy* (12%), *Annals of Surgery* (6%), *Cancer Research* (5%), and others (Table 2). It's worth noting that the *New England Journal of Medicine* published four articles, just ranked sixth in the top 10 journals with the highest EF in 2019 (0.682). Among the top 500 most frequently cited articles published from the inception of WoSCC to June 2020, the *American Journal of Gastroenterology* published the largest number.

**Table 1:** The top-10 cited articles of WoSCC bibliometrics in barrett's esophageal research.

**Rank**	**First author**	**Journal**	**Title**	**Number of citations (WoSCC)**	**Type of research**	**Type of articles**
1	Shaheen, NJ	New England journal of medicine. (2009) 360:2277–2288	Radiofrequency ablation in Barrett's esophagus with dysplasia	824	Clinical research	Article
2	Wang, KK	American journal of gastroenterology. (2008) 103:788–797	Updated guidelines 2008 for the diagnosis, surveillance and therapy of Barrett's esophagus	791	Guideline	Review
3	Hvid-Jensen, F	New England journal of medicine. (2011) 365:1375–1383	Incidence of adenocarcinoma among patients with Barrett's esophagus	759	Clinical research	Article
4	Spechler, SJ	Gastroenterology. 2011;140(3):1084–1091	American gastroenterological association medical position statement on the management of Barrett's esophagus	614	Guideline	Article
5	Winters, C	Gastroenterology. (1987) 92:118–124	Barrett's esophagus a prevalent, occult complication of gastroesophageal reflux disease	606	Clinical research	Article
6	Shaheen, NJ	Gastroenterology. (2000) 119:333–338	Is there publication bias in the reporting of cancer risk in Barrett's esophagus?	589	Review	Article
7	Sharma, P	Gastroenterology. (2006) 131:1392–1399	The development and validation of an endoscopic grading system for Barrett's esophagus: The Prague C & M Criteria	588	Clinical research	Article
8	Ronkainen, J	Gastroenterology. (2005) 129: 1825–1831	Prevalence of Barrett's esophagus in the general population: an endoscopic study	571	Comment	Article
9	Fitzgerald, RC	Gut. (2014) 63:7–42	British Society of Gastroenterology guidelines on the diagnosis and management of Barrett's esophagus	542	Guideline	Article
10	HAGGITT, RC	Human pathology. (1994) 25:982–993	Barretts-esophagus, dysplasia, and adenocarcinoma	521	Review	Review

*WoSCC, Web of Science Core Collection*.

**Table 2:** Journals with two or more of the top-100 cited articles of WoSCC bibliometrics in Barrett's esophagus.

**Rank**	**Journal**	**Number of published articles**	**Eigenfactor^TM^ score**	**Category by JCR**
1	Gastroenterology	27	0.132	Gastroenterology & Hepatology
2	American journal of gastroenterology	17	0.056	Gastroenterology & Hepatology
3	Gastrointestinal endoscopy	12	0.034	Gastroenterology & Hepatology
4	Annals of surgery	6	0.073	Surgery
4	Cancer research	5	0.181	Oncology
4	New England journal of medicine	4	0.682	General & Internal Medicine
7	Gut	4	0.065	Gastroenterology & Hepatology
7	Clinical gastroenterology and hepatology	3	0.038	Gastroenterology & Hepatology
9	Lancet	3	0.407	Medicine, General & Internal
10	Journal of thoracic and cardiovascular surgery	2	0.052	Cardiac & Cardiovascular Systems; Respiratory System; Surgery

*JCR, journal citation reports; WoSCC, Web of Science Core Collection*.

**Figure 1:**
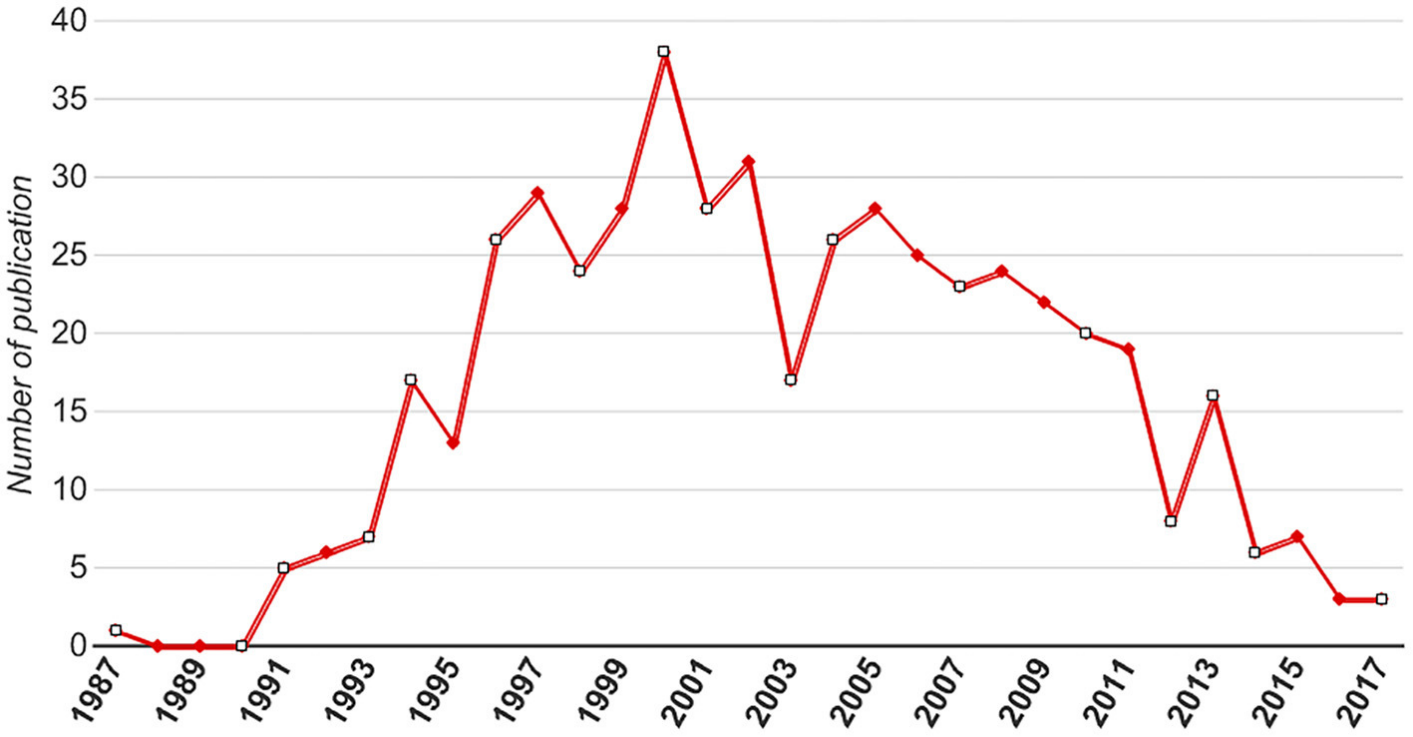
Pattern of distribution of top 500 most-cited articles (no. of article per year).

**Figure 2:**
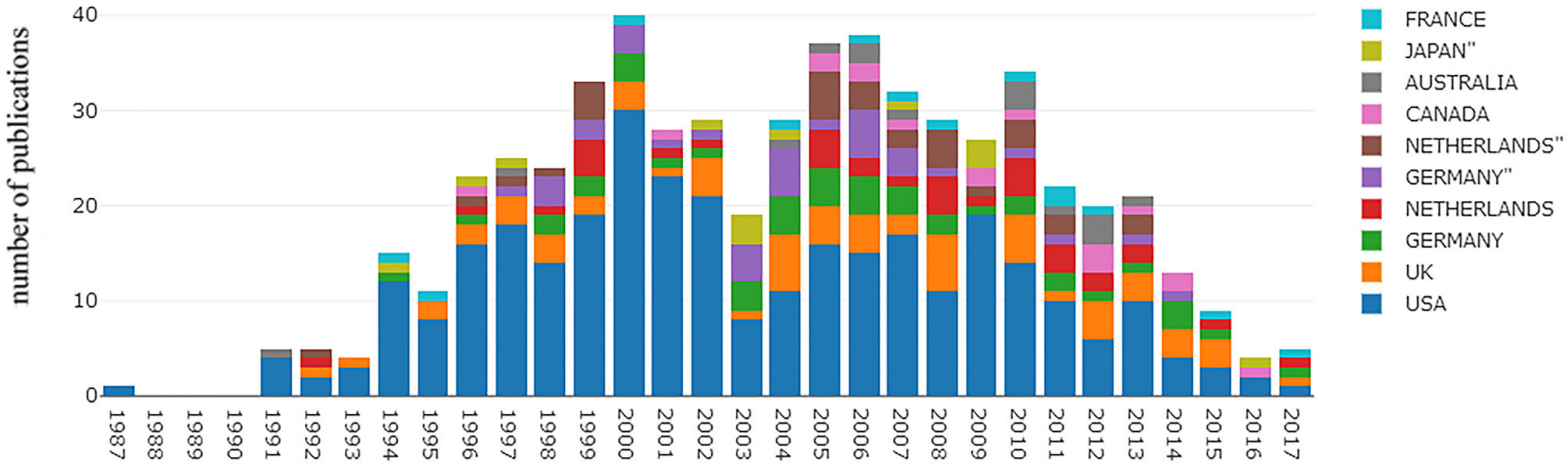
Pattern of each country' distribution of top 500 most-cited articles (no. of article per year).

The most frequently cited articles originated from several different countries (Table 3). Of the top-cited articles, the USA contributed most of the articles (*n* = 72), followed by Germany (*n* = 11), England (*n* = 10), The Netherlands (*n* = 9), Canada (*n* = 7), Australia/Japan (*n* = 5), and Belgium/North Ireland/Sweden (*n* = 4). The top 100 most frequently cited articles originated from 286 different academic institutions; the University of Washington had the highest number (12%), followed by the Cleveland Clinic (11%), and the University of Kansas/University of North Carolina/University of Southern California (7%; Table 4). Sharma Prateek and Demeester TR authored the largest number of top-100 cited articles (*n* = 6); the number of citations was 2,277 and 1,986, respectively. The additional authors are listed in Table 5. The top 500 most frequently cited articles were identified in WoSCC between 1987 and 2017 (Figure 1). There was a significant increase in the distribution of published articles worldwide before 2000. Between 1987 and 2000, there was a 38-fold increase in the number of top-cited articles related to BE. However, there has been a decline in the number of publications since 2000. In 2017, only three of the top 500 articles were published worldwide. Among these articles, the contributions from each country are shown in Figure 2. The USA was by far the leading country in BE research and contributed the majority of articles each year.

**Table 3:** Countries of origin of the top-cited articles.

**Rank**	**Countries**	**No. of articles**
1	USA	72
2	Germany	11
3	England	10
4	Netherlands	9
5	Canada	7
6	Australia	5
7	Japan	5
7	Belgium	4
9	North Ireland	4
10	Sweden	4

**Table 4:** Organizations of origin with top-100 cited articles.

**Rank**	**Organizations**	**No. of articles**
1	Univ Washington	12
2	Cleveland clin FDN	11
3	Univ Kansas	7
3	Univ n Carolina	7
3	Univ so calif	7
6	Mayo clin	6
7	Fred Hutchinson canc res ctr	6
7	Univ Amsterdam	5
9	Columbia univ	4
9	Harvard univ	4
9	Oregon hlth & sci univ	4
9	Thompson canc survival ctr	4
9	Univ Arizona	4
9	Univ calif San Francisco	4

**Table 5:** Authors with top-100 cited articles.

**Rank**	**Author**	**No. of articles**	**Citations**
1	Sharma, P	6	2,277
1	Demeester, TR	6	1,986
3	Spechler, SJ	5	1,932
3	Reid, BJ	5	1,701
3	Peters, JH	5	1,572
3	Richter, JE	5	1,430
7	Vieth, Michael	4	1,535
7	Gossner, liebwin	4	1,535
7	Rabinovitch, PS	4	1,482
7	Blount, PL	4	1,447
7	Levine, DS	4	1,235
7	Overholt, BF	4	1,162
7	Triadafilopoulos, G	4	1,122
7	Falk, GW	4	1,072

### Network Analysis Among the Top 500 Most Frequently Cited Articles in WoSCC

**Figure 3:**
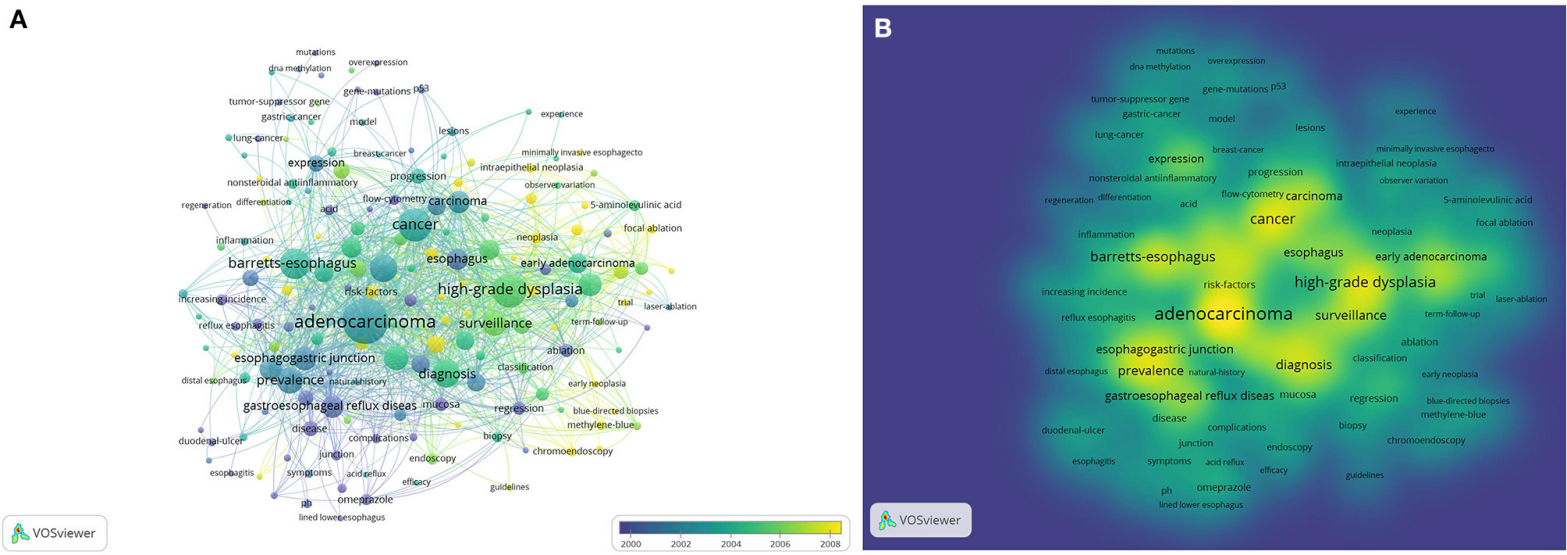
Network plot of influential keywords in barrett's esophageal research among the top-500 cited articles of Web of Science Core Collection. **(A)** overlay visualization, **(B)** density visualization of **(A)**.

To identify the influential keywords among the top 500 most frequently cited articles in WoSCC, we performed a network analysis. The units of analysis were all keywords, and a full counting method was adopted. The relevance among each word was calculated by VOSviewer. If a noun phrase appeared randomly with other noun phrases, the relevance score was low. If a noun phrase appeared primarily in conjunction with a limited number of other noun phrases, the relevance score was higher. Noun phrases with low relevance tended to be excluded, while the keywords with higher relevance typically had a more significant meaning. Figure 3A shows the overlay visualization of influential keywords in BE research. Figure 3B demonstrates the density visualization of Figure 3A. Each node represents an element analyzed. The importance of the element was judged based on the size of the node. The size of the nodes represents the keyword occurrence frequency; the larger the sphere is, the more frequently the keywords occur. Each node is colored based on its score. If the number of items around a node was larger and the weight of neighboring items was greater, the color of the node was closer to yellow. In contrast, the color of the node was closer to blue. Among the 1,363 words analyzed, “adenocarcinoma,” “high-grade dysplasia,” “cancer,” “diagnosis,” and “dysplasia” were the most influential keywords (Table 6).

**Table 6:** Keywords with the strongest occurrence among top-500 cited articles (analysis by VOSviewer).

**Rank**	**Keywords**	**Occurrences**	**Total link strength**
1	Adenocarcinoma	192	510
2	High-grade dysplasia	118	319
3	Cancer	109	301
4	Diagnosis	78	259
5	Prevalence	72	239
6	Intestinal metaplasia	62	226
7	Dysplasia	75	221
8	Esophagogastric junction	56	215
9	Surveillance	64	221
10	Gastric cardia	54	187
11	Photodynamic therapy	56	170
12	Barretts-esophagus	92	167
13	Follow-up	46	158
14	Gastroesophageal reflux disease	52	158
15	Columnar-lined esophagus	39	142
16	Risk	43	142
17	Carcinoma	55	129
18	Barrett's esophagus	47	121
19	Esophagus	48	110
20	Early adenocarcinoma	41	97

**Figure 4:**
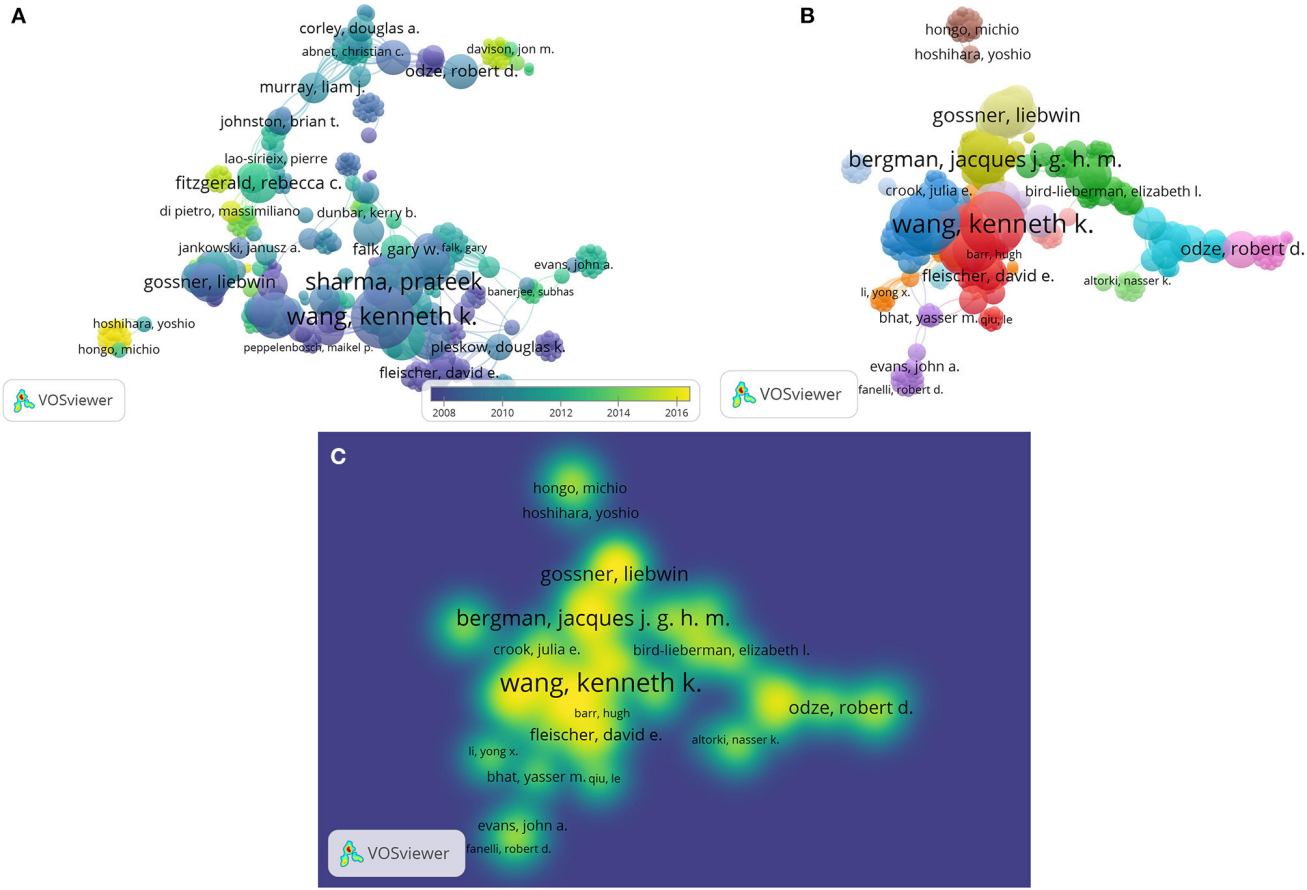
Network plot of influential authors in barrett's esophageal research among the top-500 cited articles of Web of Science Core Collection. **(A)** Overlay visualization, **(B)** visualization with LinLog layout and the modularity clustering technique of **(A)**, **(C)** density visualization of **(B)**.

To identify the influential authors among the top 500 most frequently cited articles in WoSCC, we performed a network analysis of the co-authorship. The units of analysis were authors, and a full counting method was adopted. After selecting 1 as the minimum number of documents for an author, 2,350 authors met the threshold. Among the 2,350 authors analyzed, Demeester TR was the most influential author (Table 7), ranked first among authors with top-100 cited articles. Figure 4A shows the overlay visualization of influential authors in BE research, while Figure 4B shows the visualization with LinLog layout and the modularity clustering technique of Figure 4A. Figure 4C shows the density visualization of Figure 4B.

**Table 7:** Authors with the strongest occurrence among top-500 cited articles (analysis by VOSviewer).

**Rank**	**Authors**	**Documents**	**Citations**	**Total link strength**
1	Reid, BJ	18	3,586	34
2	Galipeau, PC	11	2,150	27
3	Richter, JE	14	2,590	27
4	Sanchez, CA	11	2,076	27
5	Falk, GW	13	2,217	26
6	Rice, TW	10	1,600	25
7	Goldblum, JR	10	1,790	24
8	Demeester, TR	18	3,745	22
9	Blount, PL	10	2,341	21
10	Peters, JH	13	2,673	20
11	Demeester, SR	10	1,770	17
12	Sharma, P	10	1,647	9
13	Stolte, M	12	2,651	8
14	Vieth, M	10	2,074	8
15	Meltzer, SJ	9	1,821	6

**Figure 5:**
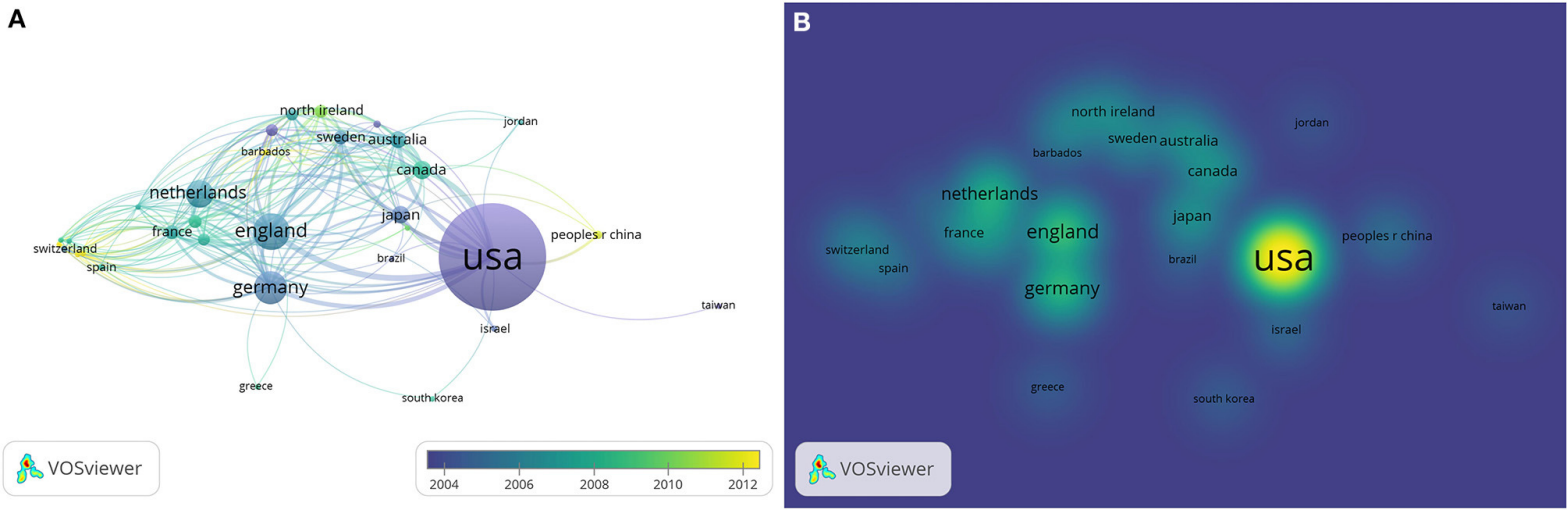
Network plot of influential countries in barrett's esophageal research among the top-500 cited articles of Web of Science Core Collection. **(A)** Overlay visualization, **(B)** density visualization of **(A)**.

To identify the influential countries among the top 500 most frequently cited articles in WoSCC, we performed a network analysis. The units of analysis were countries, and a full counting method was adopted; the most influential countries are listed in Table 8. After selecting 1 as the minimum number of documents of a country, 35 met the threshold. Figure 5A shows the overlay visualization of influential countries in BE research. Figure 5B demonstrates the density visualization of Figure 5A.

**Table 8:** Countries with the strongest occurrence among top 500 cited articles (analysis by VOSviewer).

**Rank**	**countries**	**Documents**	**Citations**	**Total link strength**
1	USA	324	60,991	110
2	England	60	12,502	90
3	Canada	21	6,093	75
4	Germany	51	8,902	61
5	Australia	17	5,165	58
6	Netherlands	39	7,807	54
7	Sweden	13	2,902	52
8	Belgium	12	3,230	44
9	North Ireland	12	2,289	43
10	Italy	10	1,578	34
11	Ireland	10	1,368	33
12	Japan	18	3,670	33
13	France	13	2,104	32
14	Scotland	10	1,434	29
15	China	6	836	7

## Discussion

It is necessary to guide decision-making in clinical practice and ongoing experimentation in a particular field to identify the impact of the medical literature. A useful index, the number of citations, has been widely adopted as an influential index for identifying the impact of an article in the medical literature database. However, even this index has been criticized, this may potentially relate to the citation of an article that arises mainly during a finite period after publication, and some influential articles may not be cited or rarely cited. An article that has been frequently cited also has great value and plays an important role in advancing knowledge in a particular field. As the widespread availability of access to the Internet has increased, the appearance of bibliometric analysis has been implemented as a method that reveals the value of an article and measures the scientific output of countries, organizations, journals, and individuals. Bibliometric analysis is widely adopted to evaluate articles in different fields of research. Compared to previous BE articles (15–17), the current study allowed us to demonstrate trends in historical development and help us to understand the prevalent areas in BE research.

Like other bibliometric articles (18–20), this analytic study provided a novel overview of the current situations and trends in BE. Besides, our study evaluated the most influential publications with a focus on BE research by using bibliometric analysis based on WoSCC. We also performed a bibliometric analysis of the top 500 cited articles and compared the results with the top 100 articles to further determine the influence of each country, institution, author, to identify research hotspots and frontier.

By identifying and characterizing the top 100 most frequently cited articles on BE, we found that the number of citations for the top 10 articles increased from 521 to 824. This number is considerably higher than exist for Helicobacter pylori (21). The top article concerning BE research had the largest number of citations of 824 times, which was far higher than the number of citations for the top-cited article about H. pylori (22). The underlying reasons for this investigation are that the presence of BE multiplies the risk of esophageal adenocarcinoma, a malignancy with a rapidly increasing incidence and poor prognosis. However, radiofrequency ablation for the treatment of dysplasia and intestinal metaplasia has a high rate of complete eradication, which not only slows the progression of dysplasia to esophageal adenocarcinoma but also decreases the occurrence of treatment-related complications (23, 24), which is of great significance for clinical treatment of BE and long-term management. Although bleeding and perforation are rare in patients requiring a long course of treatment, the most common adverse events associated with radiofrequency ablation include postoperative stenosis and postoperative chest pain. In contrast, endoscopic cryoablation, another ablation option that has emerged, has shown less postoperative chest pain and better patient tolerance than radiofrequency ablation. Although there are many new therapies, radiofrequency ablation is still the most commonly used endoscopic ablation method for BE due to its convenient use and high efficacy. Consequently, how to conquer the adverse events after radiofrequency ablation or increase the efficacy and applicability of other ablation methods may become a new research direction.

Also, the second leading article in our study, which referenced the guidelines for the diagnosis, surveillance, and therapy of BE, had 791 citations (25). The analytical results show the significance of guidelines for monitoring, diagnosis, and treatment in this area and these clinical practice guidelines have been widely used in clinical decision-making (26, 27). The purpose of monitoring is to improve patient prognosis by early detection of dysplasia to ensure effective treatment. Although there are several national and social guidelines, all of them are limited to a clear evidence base, to adequately demonstrate the benefits of monitoring. There is still debate about the target population for endoscopic surveillance, with most guidelines advocating endoscopic screening for individuals with certain risk factors, rather than for the general population, to avoid the risk and high cost of unnecessary intervention and maximize its benefits. Since endoscopic monitoring is generally considered invasive and cumbersome to perform, there is an image-based monitoring method called esophageal capsule endoscopy, which can directly and noninvasively display the esophagus, but cannot be biopsied, and is equally costly. Further studies are needed to assess the unified criteria for endoscopic monitoring of the target population and to assess the effectiveness and cost-effectiveness of various monitoring methods for different target populations.

A study included in this research on the incidence of high-grade dysplasia and esophageal adenocarcinoma in BE patients has been cited 759 times (28), ranked the third, suggesting that researchers pay attention to the progression of intestinal metaplasia into malignant tumors in BE patients with lower esophageal mucosa. The only clinical system currently used to assess the risk of BE progression is the histologic grading of dysplasia. However, some patients without dysplasia develop adenocarcinoma, and some patients with high-grade dysplasia do not. Therefore, several studies are currently attempting to establish clinical risk scores to predict the progress of BE (29). Advanced age, male, endoscopic nodular degeneration, and low-grade dysplasia have been identified as factors that increase the risk of progression. There are many other factors yet to be evaluated (30–32). Other strategies include the use of biomarkers to predict progression risk (33), but further studies are needed before they can be widely used in the clinic. The further development and improvement of the BE progression scoring system with clinical complex factors is of great significance for clinical work and management of BE.

Concerning the category by JCR of published journals, gastroenterology and hepatology are prevalent in WoSCC bibliometrics. However, journals with various categories also ranked higher in this study, such as surgery, oncology, and general and internal medicine. This finding explained that BE research is not restricted to one specific academic field and that journals with wider and various readerships can publish research on BE research as well. In the list of journals with the top 100 cited articles, Gastroenterology ranked first, with the fourth EF in 2019 (0.132) among the top 10 journals and 27 articles in the top 100, reflecting that it has been pivotal information resources. This journal was thus recognized as a fundamental research resource and played an important role in this research field. The New England Journal of Medicine ranked 6th with the highest EF in 2019 (0.682) among the top 10 journals, yet only has four articles among the top 100. The Lancet, with an EF of 0.407, ranked 9th, with only three articles among the top 100. One potential cause for this finding is that publication bias and selective reporting may result in a fewer number of publications involving BE published in some well-known journals.

In the current study, most of the top-cited articles originated from the USA. Also, most of the top 14 academic institutions conducting research on BE are located in the USA. This result reveals the overwhelming impact of the USA, not only with respect to BE research, but also on medical science research, and it may be related to the higher GDP and abundant resources (34). It has also been reported that American authors favor being partial to local articles in the citation process and American reviewers preferred articles from the US (35).

The scheme of visualization of network analysis to determine the most influential keywords, authors, and countries was increased to the top 500 cited articles. We adopted WoSCC as a biblio-metric source with in-depth analyses. We found that there were partnerships among different nations, of which the USA had the most international collaboration. As we know, globalization is a multidimensional process that includes economic, political, cultural, social, and technological-scientific aspects. Knowledge and technology can easily spread between countries, the aim of common development in an academic field makes international cooperation and knowledge exchange more significant. Although China did not have the largest number of international cooperation, the partnerships between China and the rest countries of the world, especially with the USA, are strengthening. It is worth noting that among the top 500 articles, the USA had the most publications, with the same conclusion as the top 100. Although other countries had relatively low total research production, Australia had the highest citation/article ratio, indicating that Australia has a high quality of academic output in BE research.

The network analysis among the top 500 most frequently cited articles on WoSCC showed the interest of researchers relevant to BE up to date. Keywords are highly generalized themes of the thesis, and the co-occurrence of keywords can represent research hotspots in the same period. The most influential keywords were “adenocarcinoma,” followed by “high-grade dysplasia,” “cancer,” and “diagnosis”. Besides, “risk factor,” “endoscopic mucosal resection,” “body mass index,” and “chromoendoscopy” are the current research hotspot. Similarly, visualization of network analysis of influential authors among the top 500 articles showed the extensive impact of the authors and countries. Demeester TR published 18 articles and had 3,745 citations in total and was the author with the most publications. Though Sharma Prateek ranked 5th in the top 500, he had the highest citation/article ratio, indicating the high quality of academic output in BE research. And he ranked first in the top 100, it reveals the accuracy of the analytical results and has reference significance.

The top 500 most frequently cited articles were identified in WoSCC between 1987 and 2017, and a significant increase in the distribution of published articles worldwide was observed before 2000. From 1987 to 2000, there was a 38-fold increase in the number of published articles related to BE. Among these articles, the USA was by far the predominant country in BE research and contributed most of the publications per year; however, there was a decline since 2000. In 2017 the number of most frequently cited articles on BE published worldwide was three. This finding may be potentially related to the number of citations of an article that occurs during a finite period after publication, and during this time, the number of citations increased gradually, so that some influential articles were not cited or rarely cited. It has also been reported that the true impact of an article cannot be evaluated until 20 years after its publication, and the highest number of citations per year appear 3–10 years after publication.

## Limitations

We acknowledge that there were some limitations in our study, thus the results should be interpreted with caution. The choice of using WoSCC as the only data source might limit the coverage of all possible articles. The citation searching strategy might also be insufficient because we searched these articles only by using “Barrett's esophagus,” which may have led to a lack of articles due to the use of other terminology. Since this study counted the total number of articles published in each journal, and the years of publication of these articles were different, and the EF of journals corresponding to each year was different. However, this study uniformly adopted the EF of 2019, so there were some limitations in evaluating the value of publications. Furthermore, because we carried out a quantitative co-authorship analysis, the co-authors and the cooperating counties obtained a higher rank and the rank itself did not accurately reflect the scientific quality. A corollary study is still needed in view of the deficiency of this analytic research to assess whether specific and complex searching strategies should be adopted in bibliometric analysis.

## Conclusions

In conclusion, our results revealed major advances in BE research and several hot topics over the past few decades. Our study not only provided a historical perspective on the research of BE but also helped researchers recognize the significance of the advances in this area and could serve as a reference for future academic research.

## Data Availability Statement

The original contributions presented in the study are included in the article/Supplementary Material, further inquiries can be directed to the corresponding author.

## Author Contributions

XT, ML, and XZ contributed to conception and design of the study. PG and WF organized the database. PG and HZ performed the statistical analysis. PG wrote the first draft of the manuscript. HX and CZ wrote sections of the manuscript. All authors contributed to manuscript revision, read, and approved the submitted version.

## Funding

This study is independent research funded by the following grants: Youth Foundation of Southwest Medical University (No. 0903-00031099), Doctoral research start-up funding project of Affiliated Hospital of Southwest Medical University (No. 16229), Cooperation Project of Southwest Medical University and Luzhou Government (No. 2019LZXNYDJ24), and Health Commission of Sichuan Province, Title: Practice of Adenoma-Carcinoma Sequence in Early Gastric Carcinoma Screening Projection (No. 17PJ033).

## Conflict of Interest

The authors declare that the research was conducted in the absence of any commercial or financial relationships that could be construed as a potential conflict of interest.

## Publisher's Note

All claims expressed in this article are solely those of the authors and do not necessarily represent those of their affiliated organizations, or those of the publisher, the editors and the reviewers. Any product that may be evaluated in this article, or claim that may be made by its manufacturer, is not guaranteed or endorsed by the publisher.
