# Supplementary material for: The Top-Cited Articles With a Focus on Barrett's Esophagus: A Bibliometric Analysis
Source: Front Surg. 2022 Feb 17;9:743274. doi: 10.3389/fsurg.2022.743274 (PMC8891490; doi:10.3389/fsurg.2022.743274)
Supplement: Supplementary file 1 [file Table_1.DOCX]

Supplementary Material

# Supplementary Tables

Table S1 The supporting information of the top 100 most cited articles.

| Rank | First author | Journal | Title | Number of citations(WoSCC) | Type of research | Type of articles |
| --- | --- | --- | --- | --- | --- | --- |
| 1 | Shaheen, NJ | NEW ENGLAND JOURNAL OF MEDICINE 2009;360(22):2277-2288 | Radiofrequency Ablation in Barrett's Esophagus with Dysplasia | 824 | Clinical research | Article |
| 2 | Wang, KK | AMERICAN JOURNAL OF GASTROENTEROLOGY 2008;103(3):788-797 | Updated Guidelines 2008 for the Diagnosis, Surveillance and Therapy of Barrett's Esophagus | 791 | Guideline | Review |
| 3 | Hvid-Jensen, F | NEW ENGLAND JOURNAL OF MEDICINE 2011;365(15):1375-1383 | Incidence of Adenocarcinoma among Patients with Barrett's Esophagus | 759 | Clinical research | Article |
| 4 | Spechler, SJ; | GASTROENTEROLOGY 2011;140(3):1084-1091 | American Gastroenterological Association Medical Position Statement on the Management of Barrett's Esophagus | 614 | Guideline | Article |
| 5 | Winters, C | GASTROENTEROLOGY 1987;92(1):118-124 | Barrett's Esophagus A Prevalent, Occult Complication of Gastroesophageal Reflux Disease | 606 | Clinical research | Article |
| 6 | Shaheen, NJ | GASTROENTEROLOGY 2000;119(2):333-338 | Is there publication bias in the reporting of cancer risk in Barrett's esophagus? | 589 | Review | Article |
| 7 | Sharma, P | GASTROENTEROLOGY 2006;131(5):1392-1399 | The development and validation of an endoscopic grading system for Barrett's esophagus: The Prague C & M Criteria | 588 | Clinical research | Article |
| 8 | Ronkainen, J | GASTROENTEROLOGY  2005;129(6);1825-1831 | Prevalence of Barrett's esophagus in the  general population: An endoscopic study | 571 | Comment | Article |
| 9 | Fitzgerald, RC | GUT 2014;63(1);7-42 | British Society of Gastroenterology guidelines  on the diagnosis and management of Barrett's  oesophagus | 542 | Guideline | Article |
| 10 | HAGGITT, RC | HUMAN PATHOLOGY 1994;25(10);982-993 | BARRETTS-ESOPHAGUS, DYSPLASIA, AND  ADENOCARCINOMA | 521 | Review | Review |
| 11 | Ell, C | Gastroenterology 2000;118(4):670-7 | Endoscopic mucosal resection of early cancer and high-grade dysplasia in Barrett's esophagus | 512 | Clinical research | Article; Proceedings Paper |
| 12 | Sampliner, RE | Gastroenterol 1998;93(7):1028-32 | Practice guidelines on the diagnosis, surveillance, and therapy of Barrett's esophagus | 504 | Guideline | Article |
| 13 | Reid, BJ | AMERICAN JOURNAL OF GASTROENTEROLOGY 2000;95(7):1669-76 | Predictors of progression to cancer in Barrett's esophagus: Baseline histology and flow cytometry identify low- and high-risk patient subsets | 492 | Review | Article |
| 14 | Wilson, KT | Cancer Res 1998;58(14):2929-34 | Increased expression of inducible nitric oxide synthase and cyclooxygenase-2 in Barrett's esophagus and associated adenocarcinomas | 485 | Review | Article |
| 15 | SPECHLER, SJ | Lancet 1994;344(8936):1533-6 | PREVALENCE OF METAPLASIA AT THE GASTROESOPHAGEAL JUNCTION | 481 | Clinical research | Article |
| 16 | Zhang, YW | World J Gastroenterol 2013;19(34):5598-5606 | Epidemiology of esophageal cancer | 469 | Review | Review |
| 17 | Maley, CC | Nat Genet 2006;38(4):468-73 | Genetic clonal diversity predicts progression to esophageal adenocarcinoma | 465 | Review | Article |
| 18 | Satodate, H | Gastrointest Endosc  2003;58(2):288-92 | Circumferential EMR of carcinoma arising in Barrett's esophagus: case report | 457 | Clinical research | Article |
| 19 | HINDER, RA | ANNALS OF SURGERY 1994;220(4):450-456 | LAPAROSCOPIC NISSEN FUNDOPLICATION IS AN EFFECTIVE TREATMENT FOR GASTROESOPHAGEAL REFLUX DISEASE | 450 | Clinical research | Article |
| 20 | Shaheen, NJ | Am J Gastroenterol  2016;111(1):30-50 | ACG Clinical Guideline: Diagnosis and Management of Barrett's Esophagus | 449 | Guideline | Review |
| 21 | Drewitz, DJ | Am J Gastroenterol  1997;92(2):212-5 | The incidence of adenocarcinoma in Barrett's esophagus: A prospective study of 170 patients followed 4.8 years | 438 | Review | Article |
| 22 | SPECHLER, SJ | N Engl J Med 1992;326(12):786-92 | COMPARISON OF MEDICAL AND SURGICAL THERAPY FOR COMPLICATED GASTROESOPHAGEAL REFLUX DISEASE IN VETERANS | 427 | Clinical research | Article |
| 23 | Eads, CA | Cancer Res 2001;61(8):3410-8 | Epigenetic patterns in the progression of esophageal adenocarcinoma | 426 | Review | Article |
| 24 | Spechler, SJ | N Engl J Med. 2014;371(9):836-845 | Barrett's esophagus | 419 | Review | Article |
| 25 | Schnell, TG | Gastroenterology. 2001;120(7):1607-1619 | Long-term nonsurgical management of Barrett's esophagus with high-grade dysplasia | 416 | Review | Article |
| 26 | Bhat, S | J Natl Cancer Inst. 2011;103(13):1049-1057. | Risk of Malignant Progression in Barrett's Esophagus Patients: Results from a Large Population-Based Study | 379 | Clinical research | Article |
| 27 | Ell, C | Gastrointest Endosc. 2007;65(1):3-10 | Curative endoscopic resection of early esophageal adenocarcinomas (Barrett's cancer) | 375 | Clinical research | Article |
| 28 | Overholt, BF | Gastrointest Endosc. 1999;49(1):1-7 | Photodynamic therapy for Barrett's esophagus: follow-up in 100 patients | 373 | Clinical research | Article |
| 29 | Vaezi, MF | Gastroenterology. 1996;111(5):1192-1199 | Role of acid and duodenogastroesophageal reflux in gastroesophageal reflux disease | 372 | Clinical research | Article |
| 30 | Sharma, P | Gastroenterology. 2004;127(1):310-330 | A critical review of the diagnosis and management of Barrett's esophagus: The AGA Chicago workshop | 371 | Review | Review |
| 31 | Wang, K | DIGESTION  1992;51:350-357 | EPIDEMIOLOGY AND NATURAL-HISTORY OF GASTROESOPHAGEAL REFLUX DISEASE | 350 | Clinical research | Article |
| 32 | Gossner, L | Gastroenterology 1998;114(3):448-455 | Photodynamic ablation of high-grade dysplasia and early cancer in Barrett's esophagus by means of 5-aminolevulinic acid | 348 | Clinical research | Article; Proceedings Paper |
| 33 | Shirvani, VN | Gastroenterology 2000;118(3):487-496 | Cyclooxygenase 2 expression in Barrett's esophagus and adenocarcinoma: Ex vivo induction by bile salts and acid exposure | 346 | Clinical research | Article |
| 34 | Corley, DA | Gastroenterology 2002;122(3):633-640 | Surveillance and survival in Barrett's adenocarcinomas: A population-based studyd | 344 | Clinical research | Article |
| 35 | CAMERON, AJ | Gastroenterology 1995;109(5):1541-1546 | ADENOCARCINOMA OF THE ESOPHAGOGASTRIC JUNCTION AND BARRETTS-ESOPHAGUS | 340 | Clinical research | Article |
| 36 | Overholt, BF | Gastrointest Endosc 2005;62(4):488-498 | Photodynamic therapy with porfimer sodium for ablation of high-grade dysplasia in Barrett's esophagus: international, partially blinded, randomized phase III trial | 337 | Clinical research | Article |
| 37 | Shaheen, N | JAMA  2002;287(15):1972-1981 | Gastroesophageal reflux, Barrett esophagus, and esophageal cancer - Scientific review | 334 | Review | Review |
| 38 | Kiesslich, R | Clin Gastroenterol Hepatol 2006;4(8):979-987 | In vivo histology of Barrett's esophagus and associated neoplasia by confocal laser endomicroscopy | 333 | Clinical research | Article |
| 39 | Hunter, JG | Ann Surg 1996;223(6):673-687 | A physiologic approach to laparoscopic fundoplication for gastroesophageal reflux disease | 324 | Clinical research | Article; Proceedings Paper |
| 40 | PETERS, JH | J Thorac Cardiovasc Surg 1994;108(5):813-822 | OUTCOME OF ADENOCARCINOMA ARISING IN BARRETTS-ESOPHAGUS IN ENDOSCOPICALLY SURVEYED AND NONSURVEYED PATIENTS | 323 | Clinical research | Article |
| 41 | Daly, JM | J Am Coll Surg 2000;190(5):562-573 | Esophageal cancer: Results of an American College of Surgeons patient care evaluation study | 316 | Clinical research | Article; Proceedings Paper |
| 42 | Georgakoudi, I | Gastroenterology 2001;120(7):1620-1629 | Fluorescence, reflectance, and light-scattering spectroscopy for evaluating dysplasia in patients with Barrett's esophagus | 315 | Clinical research | Article |
| 43 | van Sandick, JW | Gut 1998;43(2):216-222 | Impact of endoscopic biopsy surveillance of Barrett's oesophagus on pathological stage and clinical outcome of Barrett's carcinoma | 311 | Clinical research | Article |
| 44 | Jankowski, JA | Am J Pathol 1999;154(4):965-973 | Molecular evolution of the metaplasia-dysplasia-adenocarcinoma sequence in the esophagus | 310 | Review | Review |
| 45 | Spechler, SJ | Gastroenterology 2011;140(3):e18-e13 | American Gastroenterological Association Technical Review on the Management of Barrett's Esophagus | 308 | Review | Article |
| 46 | Gerson, LB | Gastroenterology 2002;123(2):461-467 | Prevalence of Barrett's Esophagus in asymptomatic individuals | 295 | Clinical research | Article |
| 47 | Eads, CA | Cancer Res 2000;60(18):5021-5026 | Fields of aberrant CpG island hypermethylation in Barrett's esophagus and associated adenocarcinoma | 294 | Clinical research | Article |
| 48 | Vicari, JJ | Gastroenterology 1998;115(1):50-57 | The seroprevalence of cagA-positive Helicobacter pylori strains in the spectrum of gastroesophageal reflux disease | 294 | Clinical research | Article |
| 49 | Rex, DK | Gastroenterology 2003;125(6):1670-1677 | Screening for Barrett's esophagus in colonoscopy patients with and without heartburn | 290 | Clinical research | Article |
| 50 | O'Connor, JB | Gastroenterol 1999;94(8):2037-2042 | The incidence of adenocarcinoma and dysplasia in Barrett's esophagus - Report on the Cleveland Clinic Barrett's Esophagus Registry | 290 | Clinical research | Article; Proceedings Paper |
| 51 | Buttar, NS | Gastroenterology 2001;120(7):1630-1639 | Extent of high-grade dysplasia in Barrett's esophagus correlates with risk of adenocarcinoma | 280 | Comment | Article |
| 52 | Sharma, P | Clin Gastroenterol Hepatol 2006;4(5):566-572 | Dysplasia and cancer in a large multicenter cohort of patients with Barrett's esophagus | 279 | Clinical research | Article; Proceedings Paper |
| 53 | Falk, GW | Gastroenterology 2002;122(6):1569-1591 | Barrett's esophagus | 278 | Review | Review |
| 54 | Provenzale, D | Am J Gastroenterol 1999;94(8):2043-2053 | Barrett's esophagus: A new look at surveillance-based on emerging estimates of cancer risk | 278 | Clinical research | Article |
| 55 | Barr, H | Lancet 1996;348(9027):584-585 | Eradication of high-grade dysplasia in columnar-lined (Barrett's) oesophagus by photodynamic therapy with endogenously generated protoporphyrin IX | 277 | Clinical research | Article |
| 56 | KAUER, WKH | Ann Surg 1995;222(4):525-533 | MIXED REFLUX OF GASTRIC AND DUODENAL JUICES IS MORE HARMFUL TO THE ESOPHAGUS THAN GASTRIC-JUICE ALONE - THE NEED FOR SURGICAL THERAPY RE-EMPHASIZED | 274 | Clinical research | Article; Proceedings Paper |
| 57 | Shaheen, NJ | Gastroenterology 2011;141(2):460-468 | Durability of Radiofrequency Ablation in Barrett's Esophagus With Dysplasia | 271 | Clinical research | Article |
| 58 | Rothstein, RI | Gastroenterology 1999;117(2):327-335 | Differentiation and proliferation in Barrett's esophagus and the effects of acid suppression | 271 | Clinical research | Article |
| 59 | Galanko, JA | Gastroenterol 2010;105(7):1523-1530 | Low-Grade Dysplasia in Barrett's Esophagus: Overdiagnosed and Underestimated | 268 | Clinical research | Article |
| 60 | Reid, BJ | Am J Gastroenterol 2001;96(10):2839-2848 | Predictors of progression in Barrett's esophagus II: Baseline 17p (p53) loss of heterozygosity identifies a patient subset at increased risk for neoplastic progression | 268 | Clinical research | Article |
| 61 | vanderBurgh, A | Gut 1996;39(1):5-8 | Oesophageal cancer is an uncommon cause of death in patients with Barrett's oesophagus | 266 | Clinical research | Article |
| 62 | Skacel, M | Am J Gastroenterol 2000;95(12):3383-3387 | The diagnosis of low-grade dysplasia in Barrett's esophagus and its implications for disease progression | 264 | Clinical research | Article |
| 63 | Buttar, NS | Gastroenterology 2002;122(4):1101-1112 | Chemoprevention of esophageal adenocarcinoma by COX-2 inhibitors in an animal model of Barrett's esophagus | 263 | Clinical research | Article |
| 64 | STREITZ, JM | J Thorac Cardiovasc Surg 1993;105(3):383-388 | ENDOSCOPIC SURVEILLANCE OF BARRETT-ESOPHAGUS - DOES IT HELP | 263 | Clinical research | Article |
| 65 | Sharma, P | Gastrointest Endosc 2006;64(2):167-175 | The utility of a novel narrow band imaging endoscopy system in patients with Barrett's esophagus | 259 | Clinical research | Article |
| 66 | Bennett, C | Gastroenterology. 2012;143(2):336-346. | Consensus Statements for Management of Barrett's Dysplasia and Early-Stage Esophageal Adenocarcinoma, Based on a Delphi Process | 254 | Clinical research | Article |
| 67 | Souza, RF | Cancer Res 2000;60(20):5767-5772 | Selective inhibition of cyclooxygenase-2 suppresses growth and induces apoptosis in human esophageal adenocarcinoma cells | 254 | Clinical research | Article |
| 68 | Galipeau, PC | Proc Natl Acad Sci U S A 1996;93(14):7081-7084 | 17p (p53) allelic losses, 4N (G(2)/tetraploid) populations, and progression to aneuploidy in Barrett's esophagus | 253 | Clinical research | Article |
| 69 | CLARK, GWB | Arch Surg 1994;129(6):609-614 | IS BARRETTS METAPLASIA THE SOURCE OF ADENOCARCINOMAS OF THE CARDIA | 252 | Clinical research | Article |
| 70 | Singh, SP | Cancer Res 1998;58(8):1730-1735 | Loss or altered subcellular localization of p27 in Barrett's associated adenocarcinoma | 251 | Basic science research | Article |
| 71 | Kara, MA | Gastrointest Endosc 2006;64(2):155-166 | Detection and classification of the mucosal and vascular patterns (mucosal morphology) in Barrett's esophagus by using narrow band imaging | 249 | Clinical research | Article; Proceedings Paper |
| 72 | Yousef, F | Am J Epidemiol 2008;168(3):237-249 | The incidence of esophageal cancer and high-grade dysplasia in Barrett's esophagus: A systematic review and meta-analysis | 248 | Review | Review |
| 73 | Heitmiller, RF | Ann Surg 1996;224(1):66-71 | Barrett's esophagus with high-grade dysplasia - An indication for prophylactic esophagectomy | 247 | Clinical research | Article |
| 74 | Bouma, BE | Gastrointest Endosc 2000;51(4Pt1):467-474 | High-resolution imaging of the human esophagus and stomach in vivo using optical coherence tomography | 246 | Clinical research | Article |
| 75 | Fitzgerald, RC | J Clin Invest 1996;98(9):2120-2128 | Dynamic effects of acid on Barrett's esophagus - An ex vivo proliferation and differentiation model | 244 | Review | Article |
| 76 | MIROS, M | Gut 1991;32(12):1441-1446 | ONLY PATIENTS WITH DYSPLASIA PROGRESS TO ADENOCARCINOMA IN BARRETT-ESOPHAGUS | 244 | Review | Article |
| 77 | Overholt, BF | Gastrointest Endosc 2007;66(3):460-468 | Five-year efficacy and safety of photodynamic therapy with Photofrin in Barrett's high-grade dysplasia | 241 | Clinical research | Article |
| 78 | El-Serag, HB | Am J Gastroenterol 2004;99(10):1877-1883 | Proton pump inhibitors are associated with reduced incidence of dysplasia in Barrett's esophagus | 241 | Clinical research | Article |
| 79 | WILLIAMSON, WA | Arch Intern Med 1991;151(11):2212-2216 | BARRETT-ESOPHAGUS - PREVALENCE AND INCIDENCE OF ADENOCARCINOMA | 239 | Clinical research | Article |
| 80 | Pech, O | Gastroenterology 2014;146(3):652-660 | Long-term Efficacy and Safety of Endoscopic Resection for Patients With Mucosal Adenocarcinoma of the Esophagus | 235 | Review | Article |
| 81 | Corley, DA | Gastroenterology 2007;133(1):34-311 | Abdominal obesity and body mass index as risk factors for Barrett's esophagus | 231 | Clinical research | Article |
| 82 | PROVENZALE, D | Am J Gastroenterol 1994;89(5):670-680 | A GUIDE FOR SURVEILLANCE OF PATIENTS WITH BARRETTS-ESOPHAGUS | 231 | Guideline | Article |
| 83 | Sikkema, M | Clin Gastroenterol Hepatol 2010;8(3):235-e32 | Risk of Esophageal Adenocarcinoma and Mortality in Patients With Barrett's Esophagus: A Systematic Review and Meta-analysis | 229 | Review | Review |
| 84 | Overholt, BF | Gastrointest Endosc 2003;58(2):183-188 | Photodynamic therapy for Barrett's esophagus with dysplasia and/or early stage carcinoma: long-term results | 229 | Clinical research | Article; Proceedings Paper |
| 85 | Stein, HJ | Ann Surg 2000;232(6):733-742 | Limited resection for early adenocarcinoma in Barrett's esophagus | 229 | Review | Article; Proceedings Paper |
| 86 | Hongo, M | J Gastroenterol Hepatol 2009;24(5):729-735 | Epidemiology of esophageal cancer: Orient to Occident. Effects of chronology, geography and ethnicity | 228 | Review | Review |
| 87 | Pouw, RE | Clin Gastroenterol Hepatol 2010;8(1):23-29 | Efficacy of Radiofrequency Ablation Combined With Endoscopic Resection for Barrett's Esophagus With Early Neoplasia | 227 | Clinical research | Article |
| 88 | Cameron, AJ | Am J Gastroenterol 1997;92(4):586-591 | Barrett's esophagus, high-grade dysplasia, and early adenocarcinoma: A pathological study | 225 | Clinical research | Article |
| 89 | Rastogi, A | Gastrointest Endosc 2008;67(3):394-398 | Incidence of esophageal adenocarcinoma in patients with Barrett's esophagus and high-grade dysplasia: a meta-analysis | 224 | Review | Article |
| 90 | Kara, MA | Endoscopy 2005;37(10):929-936 | High-resolution endoscopy plus chromoendoscopy or narrow-band imaging in Barrett's esophagus: a prospective randomized crossover study | 222 | Clinical research | Article |
| 91 | Spechler, SJ | N Engl J Med 2014;371(9):836-845 | Barrett's Esophagus | 221 | Review | Review |
| 92 | Panjehpour, M | Gastroenterology 1996;111(1):93-101 | Endoscopic fluorescence detection of high-grade dysplasia in Barrett's esophagus | 220 | Clinical research | Article |
| 93 | Reid, BJ | Am J Gastroenterol 2000;95(11):3089-3096 | Optimizing endoscopic biopsy detection of early cancers in Barrett's high-grade dysplasia | 219 | Clinical research | Article |
| 94 | Sherman, PM | Am J Gastroenterol 2009;104(5):1278-1296 | A Global, Evidence-Based Consensus on the Definition of Gastroesophageal Reflux Disease in the Pediatric Population | 218 | Review | Review |
| 95 | Sharma, VK | Gastrointest Endosc 2007;65(2):185-195 | Balloon-based, circumferential, endoscopic radiofrequency ablation of Barrett's esophagus: 1-year follow-up of 100 patients | 218 | Clinical research | Article; Proceedings Paper |
| 96 | Dulai, GS | Gastroenterology 2002;122(1):26-33 | Preoperative prevalence of Barrett's esophagus in esophageal adenocarcinoma: A systematic review | 217 | Review | Article |
| 97 | Lieberman, DA | Am J Gastroenterol 1997;92(8):1293-1297 | Risk factors for Barrett's esophagus in community-based practice | 214 | Review | Article |
| 98 | Falk, GW | Gastrointest Endosc 1999;49(2):170-176 | Jumbo biopsy forceps protocol still misses unsuspected cancer in Barrett's esophagus with high-grade dysplasia | 210 | Clinical research | Article; Proceedings Paper |
| 99 | Bytzer, P | Am J Gastroenterol 1999;94(1):86-91 | Adenocarcinoma of the esophagus and Barrett's esophagus: A population-based study | 209 | Clinical research | Article |
| 100 | Edelstein, ZR | Gastroenterology 2007;133(2):403-411 | Central adiposity and risk of Barrett's esophagus | 208 | Clinical research | Article |
